# Exploratory EEG Feature Profiles During Viewing of Six Combined Indoor Spatial-Color Conditions

**DOI:** 10.3390/s26185808

**Published:** 2026-09-14

**Authors:** Yingchun Li, Jiaxin Zhao, Le Ren, Rui Hong, Lingxiao Xi, Li Zhang, Xupeng Wang

**Affiliations:** 1 School of Art and Design, Xi’an University of Technology, Xi’an 710054, China; liyingchun5658@163.com (Y.L.); zhaojiaxin@stu.xaut.edu.cn (J.Z.); r1224289299@outlook.com (L.R.); 13720611073@163.com (R.H.); xilingxiao0318@163.com (L.X.); lizhang@xaut.edu.cn (L.Z.); 2 School of Mechanical and Precision Instrument Engineering, Xi’an University of Technology, Xi’an 710048, China

**Keywords:** electroencephalography, combined spatial–color condition, event-related potential, sequence-aware event mapping

## Abstract

Indoor visual environments combine room function, spatial composition, materials, and color, but these factors are often examined with subjective ratings alone. This study developed an exploratory electroencephalography (EEG) workflow for describing neural feature profiles during viewing of six non-factorial, combined indoor spatial–color conditions. Twenty adults completed three repeated runs of a counterbalanced virtual-walkthrough paradigm while 32-channel EEG was recorded at 500 Hz. Participant-specific Latin-square sequences were used to reconstruct the actual condition represented by each F1–F12 event marker. The processing archive contained 720 planned event positions, 711 unique F markers after duplicate removal, 706 epochable events, and 691 clean event-locked epochs (97.9% of epochable events). Features comprised signed mean amplitudes in literature-supported event-related-potential (ERP) windows, sustained theta/alpha/beta relative power, early/middle/late relative-power summaries, and band-limited Hilbert power-change and phase-consistency summaries. Runs were processed separately, and features were summarized within-participant before group-level description. The primary article reports no inferential results; the condition-level values, participant distributions, and waveform summaries are presented only as exploratory descriptions. The observed profiles varied across event stage, scalp region, component window, and frequency band but they do not identify a pure color or room effect, establish condition superiority, support a causal neurophysiological mechanism, or provide a current indoor-design prescription. The principal contribution is a traceable workflow for sequence-aware event mapping, quality-control accounting, and multi-domain descriptive EEG feature extraction that can inform a future prospectively powered, factorial validation study.

## 1. Introduction

Indoor environments are multidimensional visual contexts in which color, illumination, material appearance, spatial layout, and room function are perceived jointly. These features may influence subjective evaluation and physiological state, but their contributions cannot be assumed to be independent when they are embedded in complete scenes [1,2,3,4,5,6,7,8]. Color perception itself is context-dependent and shaped by learning, development, and surrounding visual information [4,5,6]. Consequently, results obtained from an intact bedroom or living-room scene should be interpreted at the level of the combined scene unless color and spatial factors have been factorially crossed.

Neuroarchitecture research has increasingly used electroencephalography (EEG), including in virtual and video-based environments, to complement self-report measures [9,10,11,12,13,14,15,16,17]. EEG can describe activity at several temporal scales. Event-related potentials (ERPs) summarize voltage changes time-locked to an event; spectral estimates characterize oscillatory activity during sustained viewing; and band-limited power and phase summaries describe additional event-locked dynamics [18,19,20,21,22,23]. Studies of indoor environments have also used EEG to examine combined environmental conditions, including spatial scale, color, thermal context, biophilic content, and illumination [24,25,26,27,28,29,30,31,32,33,34,35,36,37]. These studies motivate a multi-domain measurement strategy, but they do not justify treating any single scalp-level feature as a validated indicator of comfort, preference, or design quality.

A further methodological problem arises when event codes describe ordinal positions rather than fixed conditions. In the present experiment, F1–F12 markers denoted the initial-view and final-view events of six positions within a participant-specific Latin-square sequence. Treating F1, for example, as the same physical condition for every participant would mix different combined scenes during group averaging. A defensible analysis must therefore retain the event record, map each position to the participant’s assigned sequence, and aggregate only after the actual condition identity has been restored.

The present study was designed as an exploratory, descriptive EEG feature-profiling study. Its objective was to implement and document a sequence-aware processing workflow for six combined indoor spatial–color conditions and to summarize the resulting ERP, sustained spectral, stage-resolved spectral, and band-limited Hilbert features at the participant level. The six scenes were not a factorial crossing of room type and color; thus, the study did not test a color main effect, a room main effect, or their interaction. No inferential hypothesis testing was used, and the reported profiles are intended to generate, rather than confirm, hypotheses for future studies.

## 2. Materials and Methods

### 2.1. Participants and Analytical Sample

The final analyzable sample comprised 20 adults (12 female and 8 male participants; age 18.75 ± 0.97 years, mean ± SD). All participants were right-handed, had normal or corrected-to-normal vision, and reported no color-vision deficiency. They also reported no history of neurological or psychiatric disorders, major head trauma, or long-term medication use during the preceding six months. Participants were instructed to avoid alcohol, caffeine-containing beverages, and vigorous physical activity for at least 24 h before testing. Written informed consent was obtained before participation. Recruitment and eligibility screening were conducted in June 2026. The target of 20 participants was informed by experience from a preceding pilot and was increased for this exploratory extension rather than derived from a prospective power or precision calculation. Screening sought an approximately balanced sex distribution, normal or corrected-to-normal vision, no self-reported neurological or psychiatric disorder, geographic comparability, and broadly similar upper-body/trunk characteristics.

Each participant contributed three repeated EEG runs, yielding 60 selected recordings. In accordance with the experimental protocol confirmed by the authors, consecutive runs were separated by at least 7 days. Continuous recordings were processed separately and were not concatenated. Valid ERP and spectral run-level features were averaged both within-participant and under combined condition; Hilbert summaries were computed across the retained epochs from contributing runs, as detailed in Section 2.7. The participant, rather than the run or epoch, was therefore the observational unit for group-level description. Repeated runs may improve within-participant measurement but do not increase the independent participant sample above 20; the small and homogeneous sample therefore limits precision, reliability, and generalizability. No prospective power or precision calculation or formal statistical stopping rule was used; accordingly, the final sample of 20 participants is described as exploratory and is not presented as statistically powered to establish condition effects.

### 2.2. Laboratory Setting and EEG Equipment

The experiment was conducted in an electromagnetically shielded, light-controlled, and sound-attenuated EEG laboratory. Ambient illumination was maintained at 5 lux, viewing distance was fixed at 70 cm using a chin-and-forehead rest, and participants were asked to fixate centrally. Visual stimuli were presented on a 27-inch IPS Dell U2723QE monitor (Dell Technologies, Round Rock, TX, USA). According to the experimental record, the display was calibrated with an X-Rite i1Display Pro colorimeter (X-Rite, Incorporated, Grand Rapids, MI, USA) to D65, gamma 2.2, and a maximum luminance of 100 cd/m^2^; stimulus presentation used matched export settings across conditions.

**Figure 1:**
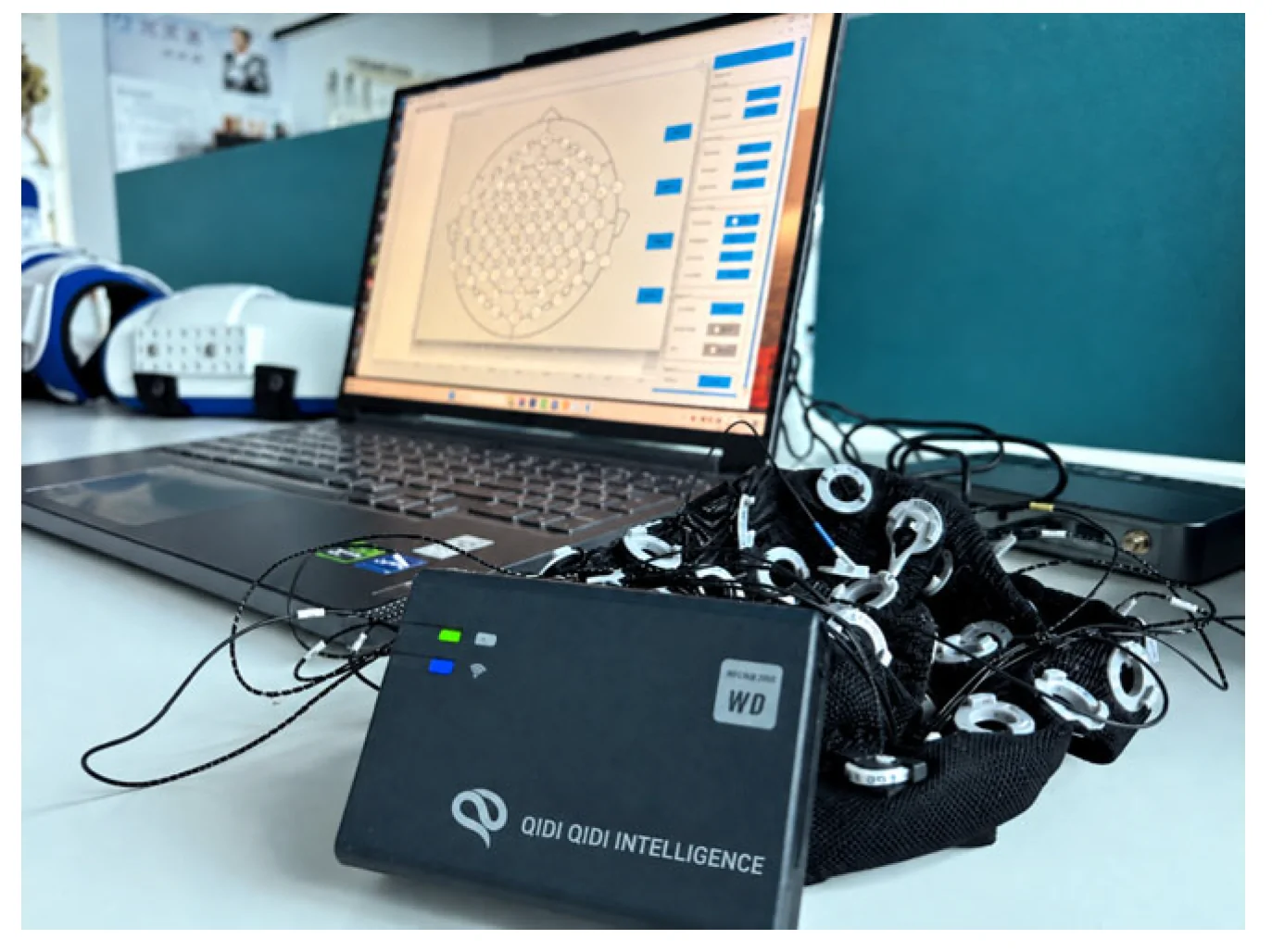
EEG acquisition system and recording interface used for 32-channel recording.

As shown in Figure 1, EEG was recorded with an iRecorderW32 wireless system (Shanghai Niantong Intelligent Technology Co., Ltd., Shanghai, China) at 500 Hz. The 32 Ag/AgCl electrodes followed the international 10–20 layout: Fp1, Fp2, AF3, AF4, F7, F3, Fz, F4, F8, FC5, FC1, FC2, FC6, T7, C3, Cz, C4, T8, CP5, CP1, CP2, CP6, P7, P3, Pz, P4, P8, PO3, PO4, O1, Oz, and O2. The nose tip served as the online reference, AFz as ground, and electrode impedances were maintained below 5 kΩ. Offline processing was performed in MATLAB R2024a with EEGLAB 2024.2.

### 2.3. Stimuli, Combined Conditions, and Sequence Mapping

**Figure 2:**
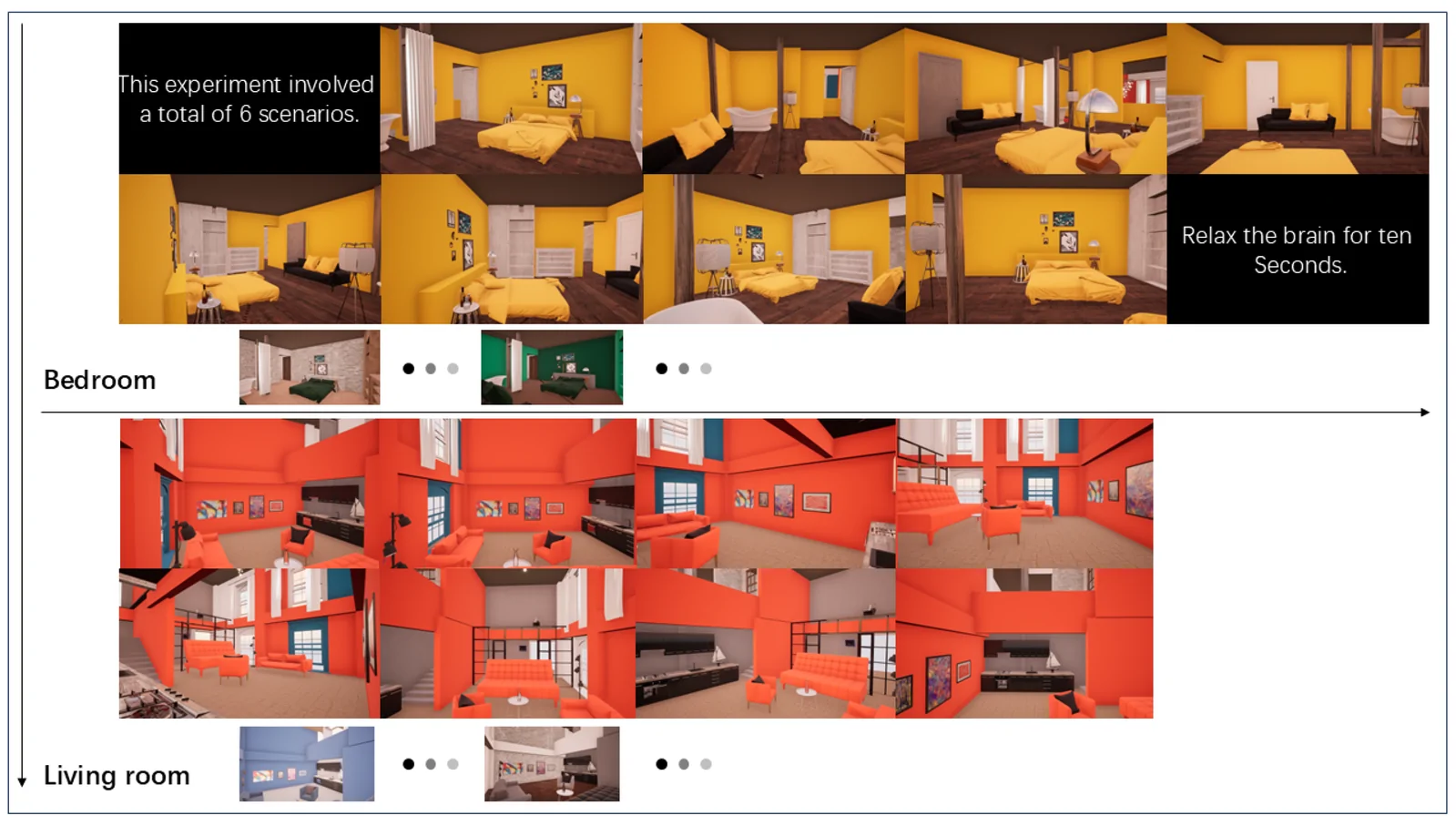
Representative synthetic indoor scenes and the event structure of the virtual-walkthrough paradigm. The scenes were controlled digital stimuli and should not be treated as direct exposure to real rooms.

The stimuli were guided virtual-walkthrough videos generated in Unreal Engine 5.5 (Epic Games, Inc., Cary, NC, USA) and exported at 1080p and 60 fps. Each stimulus video lasted 310 s. The six videos represented the following scenes: bedroom—yellow, bedroom—wood-tone, bedroom—green, living room—red, living room—blue, and living room—gray. Camera trajectories and export settings were held constant across the six videos. Because each hue or material appeared in only one room context, these stimuli constituted six combined spatial–color conditions rather than a factorial manipulation of room type and color. Any observed variation could reflect room function, layout, furnishings, material appearance, color, familiarity, or combinations of these properties, as shown in Figure 2.

Initial-view and final-view event markers delimited each condition segment. Instruction screens and 10 s blank intervals separated successive conditions. Sustained features were computed only between an available paired initial-view and final-view marker, excluding 0.5 s at each edge. Each valid sustained segment was also divided into three equal-duration parts labeled early, middle, and late. The final-view marker corresponded to the first displayed frame of the new view designated as the final-view event. Its recorded event timestamp defined t = 0. Final-view epochs therefore characterize the EEG response to the appearance of that view rather than an offset-only or blank-interval response.

**Figure 3:**
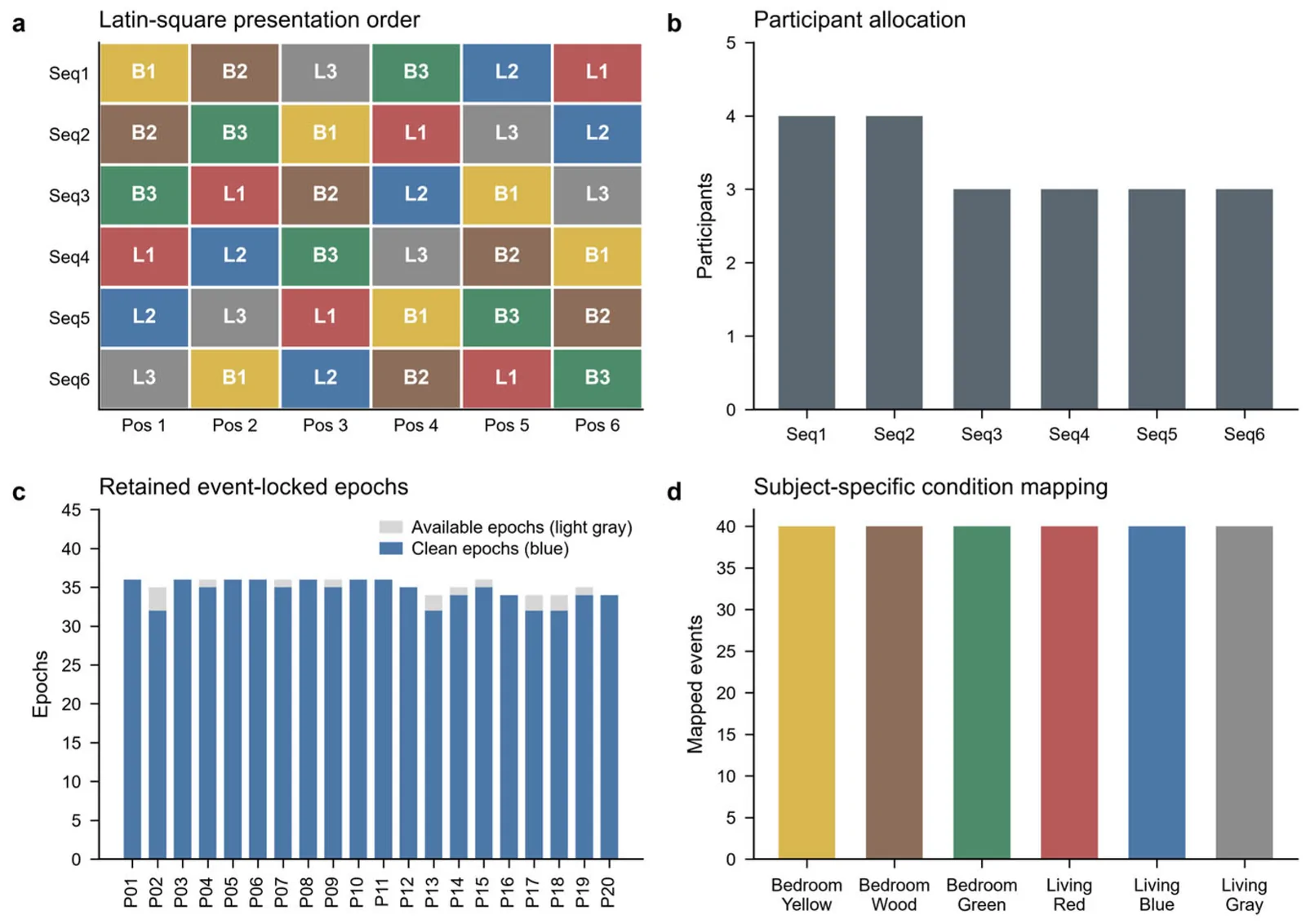
Counterbalancing and mapping. (**a**) Sequence order; (**b**) participant allocation; (**c**) available (light gray) and retained clean (blue) epochs; (**d**) mapped events by condition.

Stimulus order was counterbalanced with six Latin-square sequences. Conditions were coded B1 (bedroom—yellow), B2 (bedroom—wood-tone), B3 (bedroom—green), L1 (living room—red), L2 (living room—blue), and L3 (living room—gray). Sequence assignments were Seq1, B1–B2–L3–B3–L2–L1 (P01–P03 and P19); Seq2, B2–B3–B1–L1–L3–L2 (P04–P06 and P20); Seq3, B3–L1–B2–L2–B1–L3 (P07–P09); Seq4, L1–L2–B3–L3–B2–B1 (P10–P12); Seq5, L2–L3–L1–B1–B3–B2 (P13–P15); and Seq6, L3–B1–L2–B2–L1–B3 (P16–P18). F1/F2 represented the initial/final events of sequence position 1, F3/F4 those of position 2, and so forth through F11/F12 for position 6. Event codes were mapped to combined conditions separately for each participant before averaging, as shown in Figure 3.

### 2.4. Preprocessing and Quality Control

**Figure 4:**
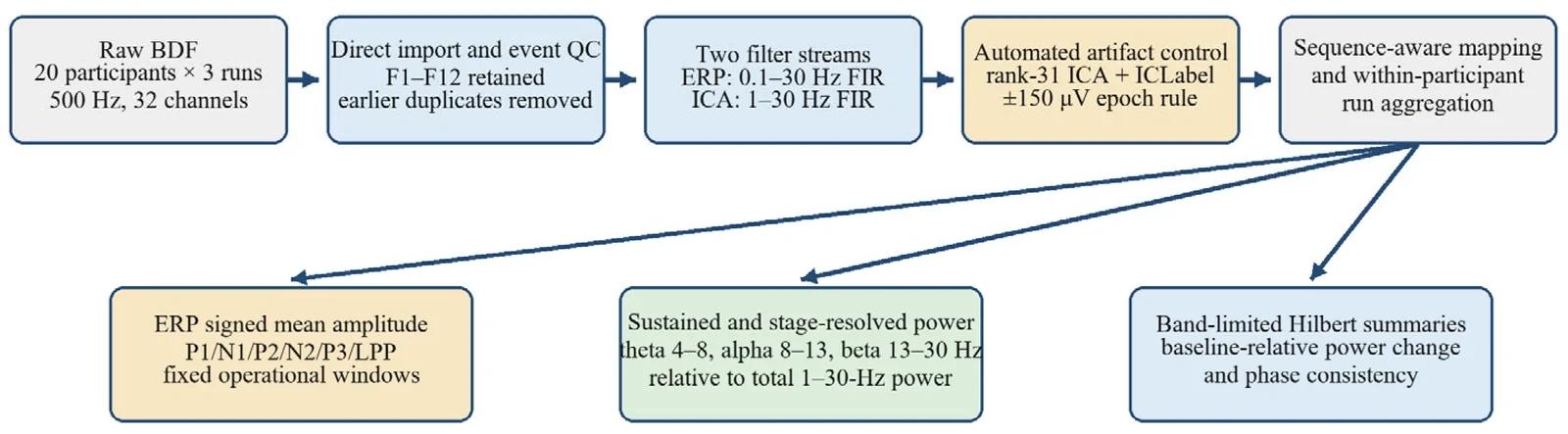
Executed preprocessing, quality-control, sequence-mapping, and feature-extraction workflow. The diagram contains only operations documented in the final raw-rerun branch; Section 2.4 reports the implementation details and decision thresholds.

Raw bdf files were imported directly into MATLAB R2024a using the EEGLAB 2024.2 eConScan importer; no intermediate data-format conversion was performed. F1–F12 event markers were preserved, and earlier duplicate F-marker occurrences were removed so that the final occurrence was retained. EEGLAB consistency checks were applied after import and key transformations. The final raw-rerun branch did not implement resampling, notch filtering, automated bad-channel detection or interpolation, artifact subspace reconstruction, or manual epoch rejection. Two filtered data streams were created. The ERP stream used EEGLAB pop_eegfiltnew with a zero-phase, non-causal Hamming-windowed sinc finite-impulse-response band-pass filter from 0.1 to 30 Hz (order 16,500; 16,501 coefficients; transition width 0.1 Hz; −6 dB cutoffs 0.05 and 30.05 Hz). The independent-component-analysis (ICA) stream used the same implementation from 1 to 30 Hz (order 1650; 1651 coefficients; transition width 1 Hz; −6 dB cutoffs 0.5 and 30.5 Hz). Both streams were re-referenced to the average reference. Extended runica was fitted to the 1–30 Hz stream at rank 31, and the resulting weights were transferred to the 0.1–30 Hz ERP stream. ICLabel classification was fully automated. A component was rejected when the maximum probability among Muscle, Eye, Heart, Line Noise, and Channel Noise was at least 0.80 and its Brain probability was below 0.30. Across 60 runs, 139 of 1860 components were removed (119 classified primarily as Eye, 16 as Muscle, three as Channel Noise, and one as Heart); no expert visual adjudication was documented. Event-locked epochs extended from −200 to 800 ms around each available F marker and were baseline-corrected to −200 to 0 ms. An epoch was automatically marked for rejection if the absolute voltage at any sample in any retained EEG channel exceeded 150 µV anywhere within the complete −200 to 800 ms epoch; no additional manual epoch rejection was performed. The same filtering, ICA transfer, epoch interval, baseline, rejection rule, ROI definitions, feature windows, and aggregation procedure were applied to initial-view and final-view events. Sequence-aware condition mapping was completed before within-participant averaging. Figure 4 summarizes this executed route.

### 2.5. ERP Features

Three regions of interest (ROIs) were defined in the executed MATLAB script: occipital (O1, Oz, O2), parietal (P3, Pz, P4), and frontocentral (Fz, Cz, C3, C4). Channels within each ROI were averaged before feature measurement. ERP features were quantified as signed mean voltage, in microvolts, within one set of operational windows applied identically to every participant, run, combined condition, and event stage: P1, 80–130 ms; N1, 130–200 ms; P2, 160–240 ms; N2, 250–350 ms; P3, 300–600 ms; and late positive potential (LPP), 500–800 ms. All six windows were computed for each ROI; the primary descriptive displays used occipital P1/N1, parietal P3, and frontocentral late-window amplitude (the operational LPP window). The P1 and N1 intervals are supported by [38], P2 by [39], N2 by [40], P3 by [41], and LPP by [42]. These sources support latency plausibility only; the component names denote operational measurement windows and do not establish canonical morphology, component identity, or a specific neural mechanism in the present data.

### 2.6. Sustained and Stage-Resolved Spectral Features

For each available initial/final event pair, the signal between 0.5 s after the initial marker and 0.5 s before the final marker was averaged within ROI and linearly detrended. Power spectral density was estimated using Welch’s method with a Hamming window of up to 2 s, 50% overlap, and a fast-Fourier-transform length equal to the next power of two at or above the window length. For a full 2 s window at 500 Hz (1000 samples), NFFT was 1024, corresponding to a frequency-bin spacing of 500/1024 = 0.4883 Hz; shorter edge-limited windows followed the same next-power-of-two rule and could have different spacing. Absolute power was integrated for theta (4–8 Hz), alpha (8–13 Hz), beta (13–30 Hz), and the total 1–30 Hz range. Relative power was the band power divided by total 1–30 Hz power. The same calculation was repeated within the early, middle, and late thirds of each valid sustained segment.

### 2.7. Band-Limited Hilbert Summaries

Event-locked ROI data were additionally filtered into theta (4–8 Hz), alpha (8–13 Hz), and beta (13–30 Hz) bands with a fourth-order Butterworth band-pass filter applied forward and backward using filtfilt. The analytic signal was obtained with the Hilbert transform. Epoch-wise instantaneous power was expressed relative to the −200-to-0 ms baseline as 10 × log10 [post-event power/baseline power]. Mean power change was summarized over 200–600 ms for theta, 200–800 ms for alpha, and 300–800 ms for beta. Within each participant, condition, and event stage, channels were averaged within each ROI and retained epochs from contributing runs were pooled as separate epoch columns. Filtering and Hilbert transformation were applied separately to each epoch. At each time point, phase consistency was the magnitude of the mean unit phasor across epochs; that magnitude was then averaged over the band-specific window. It was not an average of single-run phase-consistency values. Power change was averaged over the window and retained epochs. Group summaries used unweighted means of available participant-level values, omitting undefined phase-consistency values. These outputs are fixed-band, fixed-window Hilbert summaries rather than full wavelet-based or newtimef time–frequency maps. Each participant/event cell contained only one to three valid epochs; phase consistency was undefined for one-epoch cells and is sensitive to small and unequal trial counts. It was therefore retained only as an exploratory descriptive output. Wavelet type, cycle count, and two-dimensional time–frequency resolution are not applicable because no wavelet, short-time Fourier, or newtimef decomposition was executed. The absolute phase-consistency magnitude is not evidence of meaningful neural phase locking, is not quantitatively comparable with conventional many-trial ITC, and has not been shown to be stable in this low-trial design. Exact participant-by-condition-by-stage counts, event dispositions, and contributor denominators are reported in Supplementary Data S1.

### 2.8. Descriptive Analysis and Reporting Boundary

ERP and spectral run-level features were averaged within-participant for each applicable combined condition, event stage, ROI, and feature; Hilbert summaries were computed across retained epochs as described in Section 2.7. Group summaries were then calculated from participant-level values. Figures show individual participant values, group means, descriptive standard errors where available, or condition-level heatmaps. The primary article remains descriptive: no inferential results, *p*-values, multiple-comparison procedures, confidence intervals, effect sizes, or claims of statistical significance are reported or used to support condition effects. Numerical contrasts are reported as observed ranges or profiles and are not interpreted as evidence that conditions differ beyond within-participant variability or measurement noise. The present report is therefore deliberately neurophysiology-only and does not interpret EEG features as direct measures of comfort, preference, emotion, or performance.

## 3. Results

### 3.1. Event Accounting and Retained Data

The planned design contained 20 participants × 3 runs × 6 combined conditions × 2 event positions = 720 event positions. The selected BDF files contained 719 F-marker occurrences. Removing eight earlier duplicate occurrences left 711 unique F markers, meaning that nine planned markers were absent. Five of the 711 markers were F12 events too close to the recording boundary to form a complete −200 to 800 ms epoch, leaving 706 epochable events. Fifteen epochable events exceeded the automated ±150 µV criterion, leaving 691 clean epochs. The clean-epoch retention rate was therefore 691/706 = 97.9%; it was not calculated against the 720 planned positions. The acquisition-level cause of the nine absent markers could not be recovered from the archive, as shown in Table 1.

Participant-level clean-epoch totals ranged from 32 to 36 (mean 34.55, SD 1.50). Thus, all 20 participants contributed data, but valid event availability was not identical across participants, event stages, or combined conditions. Of the 240 participant-by-condition-by-stage cells, 214 contained three clean epochs, 23 contained two, and three contained one. The three one-epoch cells were the bedroom-green final-view cells for P16, P17, and P18; their phase-consistency values were undefined and excluded, not set to zero. Stage-specific phase-consistency means therefore had 17 contributors for bedroom-green final-view and 20 for every other condition-stage cell; power-change means 20 throughout. Supplementary Data S1 provides the complete ledger and reconciles missing markers, boundary-incomplete events, and amplitude-based rejection.

### 3.2. ERP Waveform and Window Summaries

**Figure 5:**
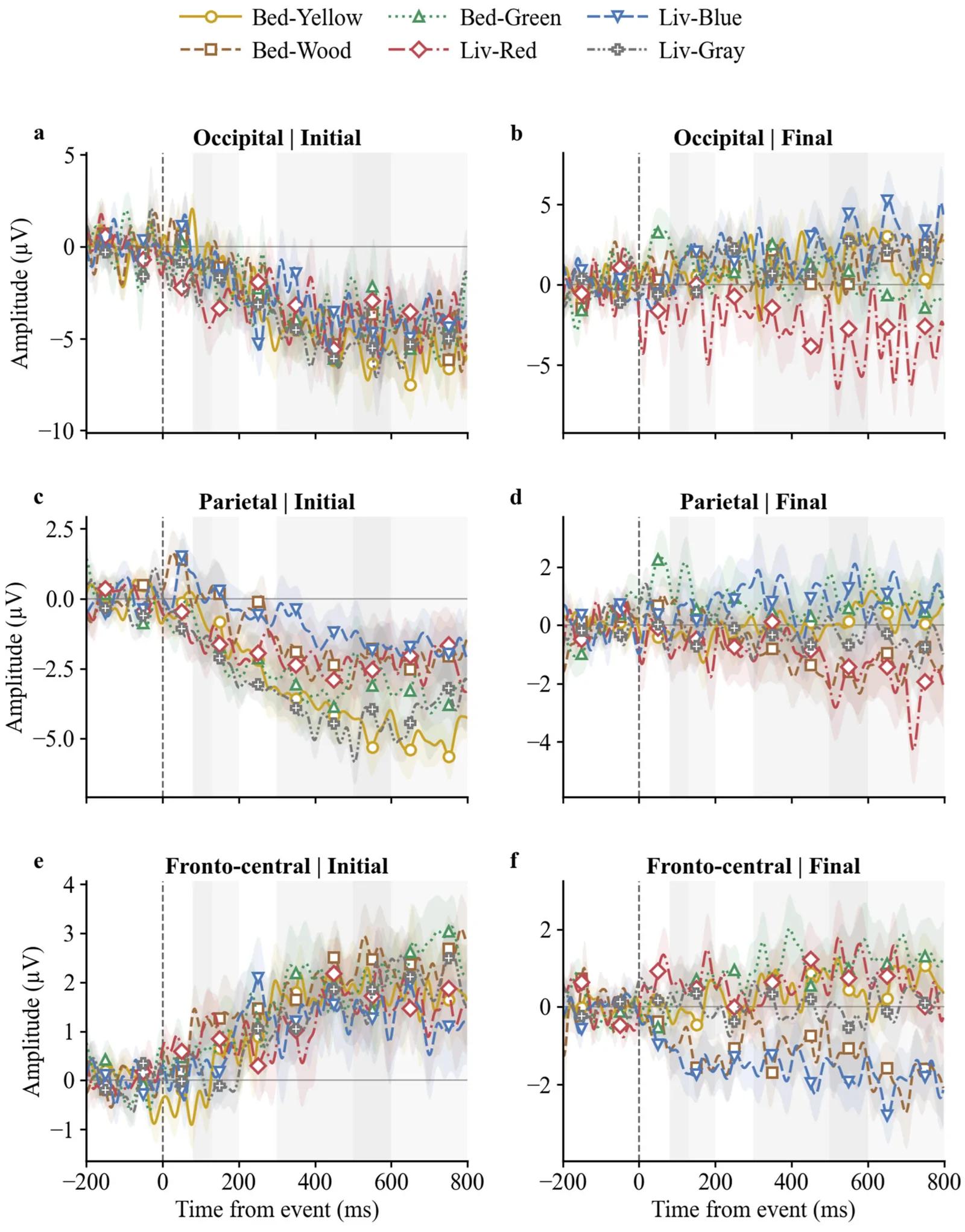
Group means of participant-level ERP waveforms (n = 20) for the six combined conditions: (**a**) occipital ROI, initial-view events; (**b**) occipital ROI, final-view events; (**c**) parietal ROI, initial-view events; (**d**) parietal ROI, final-view events; (**e**) frontocentral ROI, initial-view events; (**f**) fronto-central ROI, final-view events. Lines show group means of participant-level waveforms; ribbons show descriptive SEM across participants; shading identifies the P1, N1, P3, and LPP operational windows. The six conditions are distinguished by condition-matched colors, line types, and markers. No smoothing or inferential comparison was applied. Visual separation among curves is not evidence of a condition effect.

Figure 5 separates the initial-view and final-view waveforms for each ROI. The initial-view occipital and parietal averages showed a broad negative shift after approximately 200 ms, whereas the frontocentral initial-view averages were more positive over later latencies. Final-view waveforms were more variable in sign and morphology across conditions and ROIs. These observations describe the plotted averages only. Identical preprocessing and operational windows do not establish physiological equivalence between the two event stages; final-view waveforms are interpreted only as responses time-locked to the first displayed frame of the new final view, without assigning a unique cognitive process or a video-offset mechanism.

**Figure 6:**
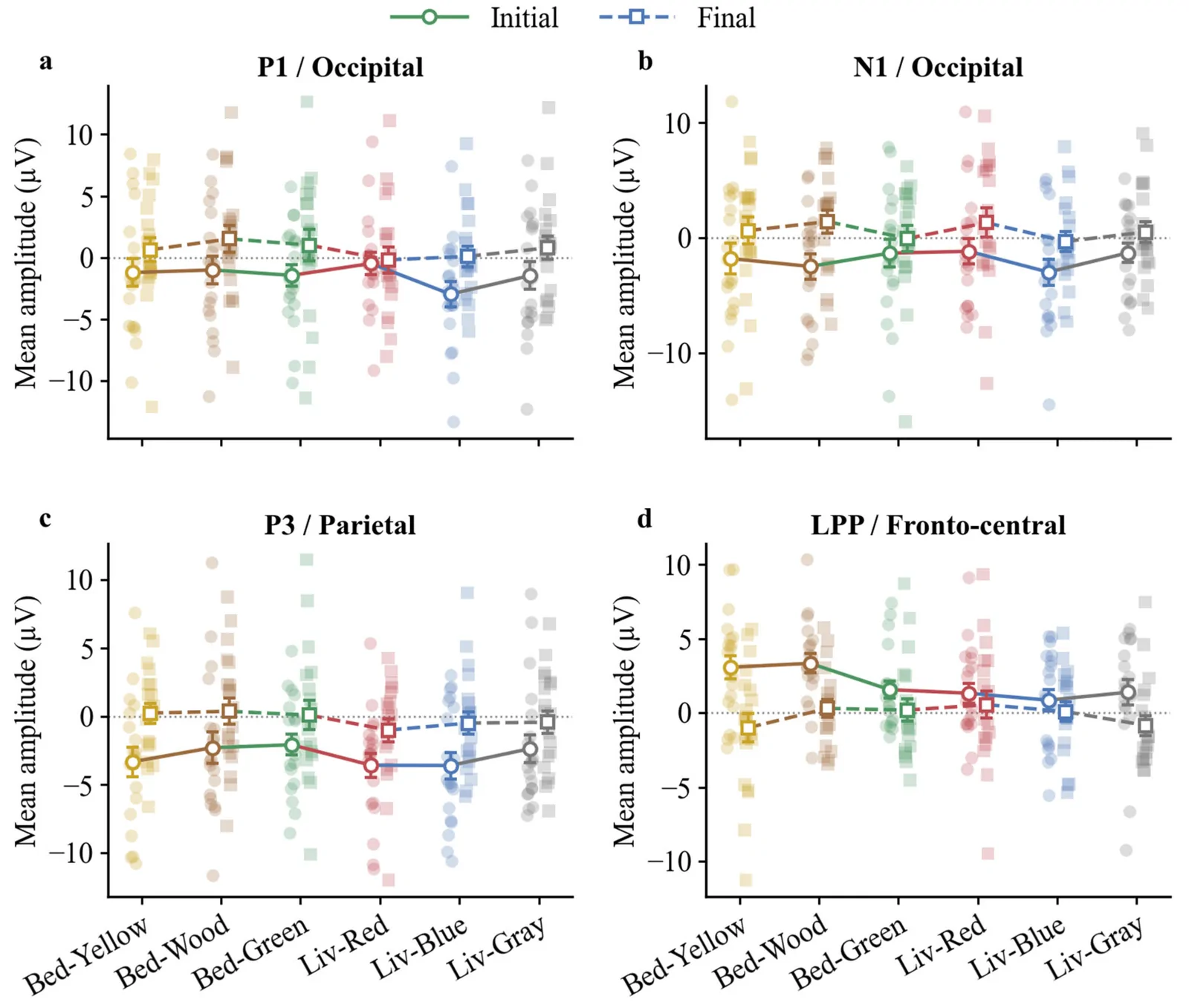
Participant-level signed mean amplitudes for four window/ROI summaries: (**a**) occipital P1; (**b**) occipital N1; (**c**) parietal P3; (**d**) frontocentral late window (500–800 ms; operational LPP window). Pale condition-colored points show participant values; condition-matched colored markers and connecting segments show group means with descriptive SEM for initial-view (circles/solid line) and final-view (squares/dashed line) events. The participant, not the run or epoch, is the observational unit. Participant overlap and dispersion are descriptive and do not establish condition effects.

Participant distributions for the four primary window/ROI summaries are shown in Figure 6. When values were collapsed in the condition-level descriptive summaries, mean occipital P1 amplitudes ranged from −1.446 to 0.250 µV, parietal P3 amplitudes from −2.311 to −0.981 µV, and frontocentral late-window amplitudes (500–800 ms) from 0.264 to 1.812 µV. These ranges are small relative to the visible participant dispersion.

### 3.3. Sustained Relative-Power Profiles

**Figure 7:**
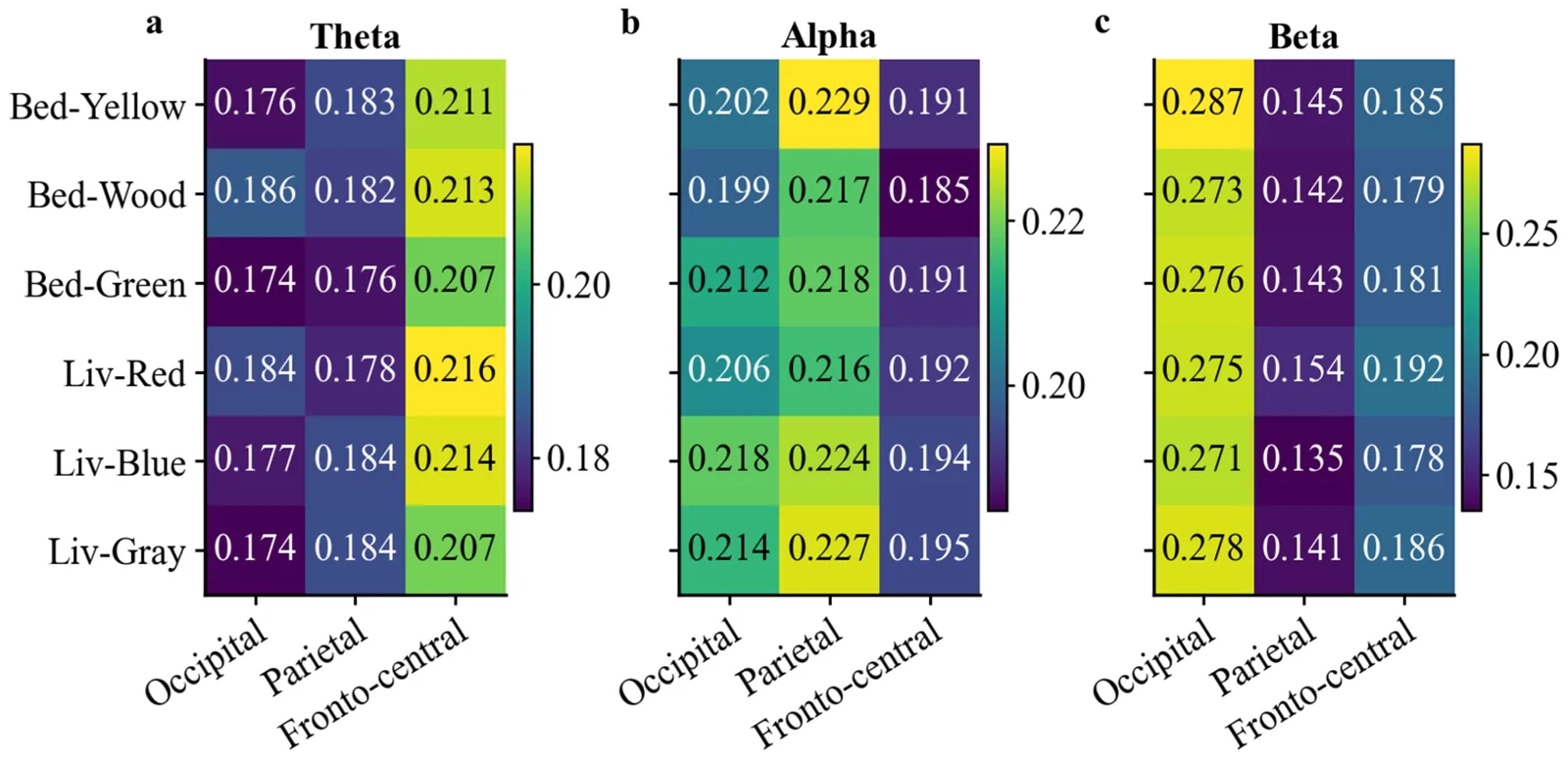
Condition-level means of participant-level (*n* = 20) theta (4–8 Hz), alpha (8–13 Hz), and beta (13–30 Hz) relative power during sustained viewing: (**a**) theta, (**b**) alpha, and (**c**) beta. Each band was normalized by total 1–30 Hz power; cell values are descriptive means. The displayed numerical differences are not inferential evidence of condition effects.

Figure 7 documents sustained relative power by band, ROI, and combined condition. Across the six displayed sets of condition-level means, the heatmaps show a recurring descriptive ROI-by-band organization, but no condition ranking or systematic condition effect is established. These are scalp-level descriptive distributions and were not used to infer a unique cognitive process. In the condition-level summaries used in the original feature report, alpha relative-power means spanned 0.200–0.212 and beta means spanned 0.195–0.207; the narrow ranges are reported without inferential interpretation.

### 3.4. Stage-Resolved and Hilbert Summaries

**Figure 8:**
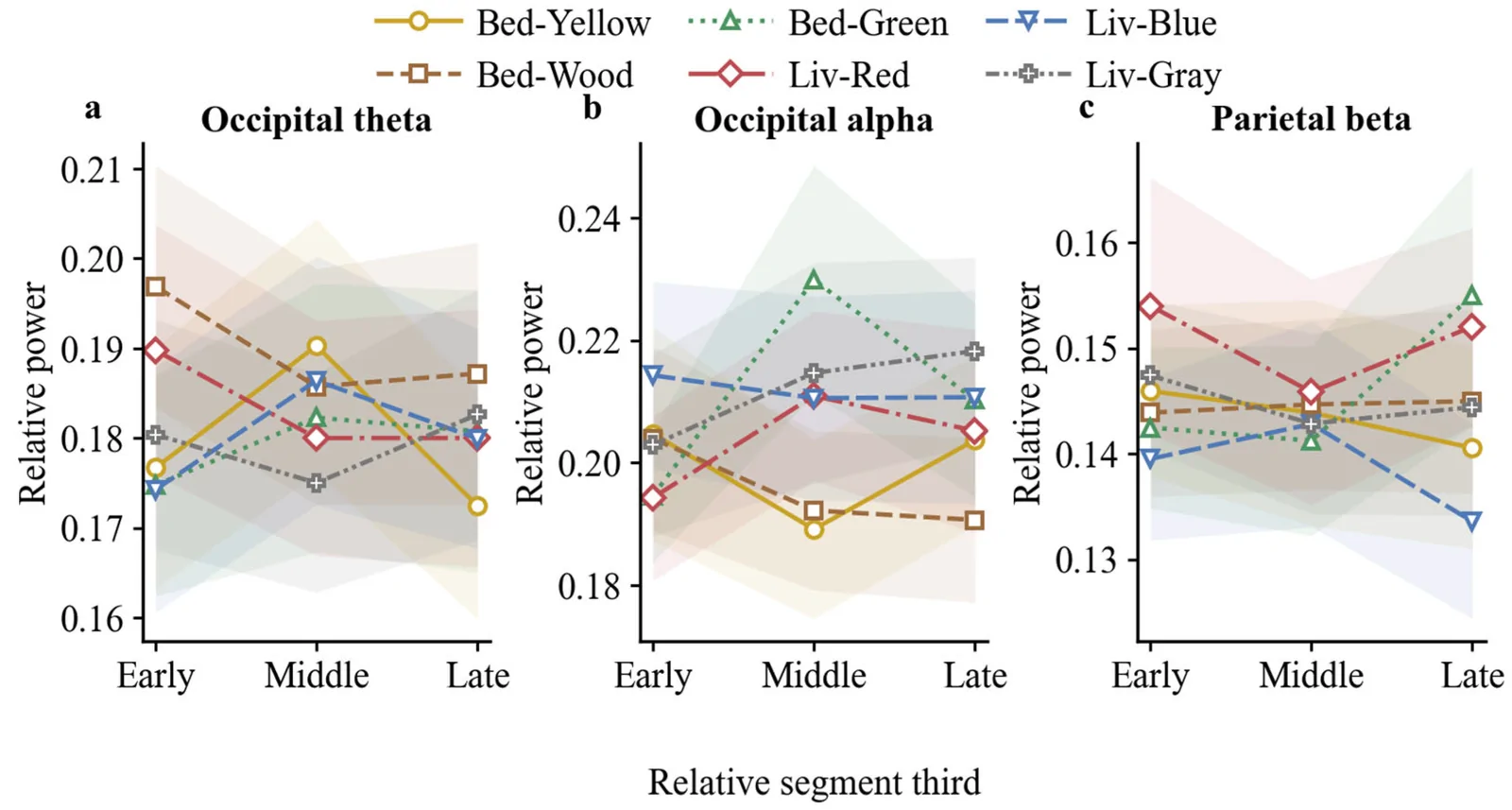
Descriptive early-, middle-, and late-third relative-power trajectories for (**a**) occipital theta, (**b**) occipital alpha, and (**c**) parietal beta. Each of the six conditions is distinguished by a condition-matched color, line type, and marker; ribbons show descriptive SEM across participants (*n* = 20). Stage labels represent relative segment thirds, not fixed durations. The trajectories do not establish condition effects.

The early/middle/late trajectories were not uniformly monotonic (Figure 8). For example, some condition traces increased in the middle third and returned toward their initial value in the late third, whereas others changed in the opposite direction. Because the three stages were relative thirds of segments that could differ in duration, these trajectories are descriptive within-segment summaries rather than evidence for a common absolute-time process. The aggregate condition means across the selected temporal summaries occupied a narrow range (0.199–0.203). No systematic condition effect or common temporal mechanism is inferred from these trajectories.

Band-limited Hilbert power-change and phase-consistency values are retained only as supplementary technical documentation. In the collapsed condition summaries, mean power change ranged from −3.000 to −2.498 dB and mean phase consistency from 0.531 to 0.557. Absolute phase-consistency magnitude is not evidence of meaningful neural phase locking, is not quantitatively comparable with conventional many-trial ITC, and does not support condition comparison because only one to three epochs contributed per participant/event cell and one-epoch cells were undefined. Stability was not established. The detailed band-by-ROI heatmaps remain in Supplementary Figure S1 rather than the primary results. The descriptive scope and interpretation limits of the four analytical domains are summarized in Table 2.

## 4. Discussion

### 4.1. Principal Contribution and Interpretation Boundary

The main contribution of this study is methodological and descriptive: it reconstructs the true condition identity of ordinal event codes, documents the event-loss chain, applies one executed preprocessing route, and summarizes several EEG feature domains after within-participant aggregation. This sequence-aware mapping is essential because the Latin-square design assigned different physical scenes to the same ordinal F code across participants. The workflow prevented condition mixing that would otherwise arise from averaging solely by event label.

The numerical summaries are indexed by event stage, ROI, latency window, frequency band, and segment position. This multi-domain organization is useful for exploratory description, but it does not demonstrate systematic condition effects. It does not show that one combined condition is neurophysiologically superior or that the observed ranges exceed within-participant variability or measurement noise. The six stimuli were compound, non-factorial scenes; hence, none of the reported values can isolate a causal contribution of color, room function, material appearance, furnishings, or spatial semantics.

### 4.2. ERP Windows and Waveform Morphology

The operational component windows, relative to event onset, were P1 (80–130 ms), N1 (130–200 ms), P2 (160–240 ms), N2 (250–350 ms), P3 (300–600 ms), and LPP (500–800 ms); these intervals fall within published visual ERP latency ranges [38,39,40,41,42]. Applying the same numerical windows to every participant and condition improves measurement consistency, but a latency label alone does not demonstrate that each grand-average waveform has canonical component morphology. This distinction is especially important for the broad negative shifts after approximately 200 ms in some initial-view averages. The same numerical windows and preprocessing were applied to both event stages only for measurement consistency; they do not imply that initial-view and final-view responses are physiologically equivalent. The final-view waveform is time-locked to the first frame of the newly appearing final view and describes the EEG response to that visual appearance; this operational definition does not establish a unique cognitive mechanism.

### 4.3. Sustained and Stage-Resolved Spectral Descriptions

The sustained-power heatmaps exhibited recognizable ROI-by-band structure, while condition-level mean ranges were comparatively narrow. Existing literature links oscillatory EEG activity to visual attention and environmental experience [15,16,17,24,25,26,27,28,29,30,31,32,33,34,35,36,37], but those functional associations are task- and analysis-dependent. Dividing each paired segment into equal thirds retained within-segment temporal information that would be lost in a single whole-segment mean. However, the early, middle, and late labels were relative to each segment, not aligned fixed-duration experimental periods. Apparent bends or crossings in the trajectories should therefore be treated as candidate patterns for future confirmation, not as demonstrated phases of neural regulation.

### 4.4. Hilbert Power and Phase Summaries

The archived analysis used fourth-order forward/backward Butterworth filtering and a Hilbert analytic signal to obtain fixed-band, fixed-window power changes and phase consistency. It did not calculate a wavelet, short-time Fourier, newtimef, or other full time–frequency decomposition; wavelet type, cycle count, and two-dimensional resolution are therefore not applicable. Phase consistency estimated from one to three epochs is susceptible to substantial finite-sample and unequal-trial-count bias. Its absolute magnitude is not evidence of meaningful neural phase locking, is not comparable quantitatively with conventional many-trial ITC, and was not shown to be stable. The summary documents what was computed but is unsuitable for condition ranking or mechanistic interpretation. A future study should increase artifact-free trials, prespecify bands and windows, equalize or model trial counts, and use a fully documented time–frequency method if a time-resolved claim is intended.

### 4.5. Limitations and Future Work

Several limitations define the scope of the findings. The independent sample contained only 20 young adults with a narrow age distribution; repeated runs do not increase that sample, and no prospective power or precision calculation or formal statistical stopping rule was used. Precision, reliability, and population generalizability are therefore limited. The design did not factorially cross room type, hue, material, lighting, and layout, so textual revision cannot resolve the confound and pure factor effects or interactions are not identifiable. Synthetic screen-presented walkthroughs improved experimental control but did not reproduce multisensory, embodied exposure to real rooms. ICLabel decisions were fully automated and no expert visual adjudication was documented; residual artifact and automated misclassification remain possible. Although exact trial counts and contributor denominators are documented in Supplementary Data S1, one to three epochs per cell remain insufficient to establish phase-consistency stability or meaningful neural phase locking. These limitations preclude current architectural or environmental-design guidance.

Future validation should use a larger prospectively planned sample, a fully crossed stimulus design, more artifact-free trials, and predefined primary outcomes. Deeper participant-level statistical analysis should be prespecified and should include appropriate repeated-measures or mixed-effects framework, multiplicity control, effect sizes, uncertainty intervals, assumption checks, and sensitivity analyses. Future work should also integrate traceably mapped subjective and behavioral outcomes, test preprocessing sensitivity, and compare controlled digital scenes with immersive or real-space exposure.

## 5. Conclusions

This exploratory study provides a traceable workflow for sequence-aware event mapping, EEG quality-control accounting, and participant-level extraction of ERP, sustained spectral, stage-resolved spectral, and band-limited Hilbert features during viewing of six combined indoor spatial–color scenes. The verified event chain was 720 planned positions, 711 unique markers, 706 epochable events, and 691 clean epochs. The primary article presents descriptive feature profiles and does not use inferential results to support condition effects. With only 20 young adults and compound non-factorial scenes, no pure color or room effect, condition superiority, causal mechanism, broad population generalization, or current indoor-design prescription is supported. The workflow and observed ranges can inform a larger, prospectively planned factorial study with deeper prespecified statistical analysis.

## Figures and Tables

**Table 1:** Event-locked epoch accounting by combined condition.

Combined Condition	Epochable	Rejected	Clean
Bedroom—yellow	119	5	114
Bedroom—wood-tone	118	1	117
Bedroom—green	114	3	111
Living room—red	117	4	113
Living room—blue	119	2	117
Living room—gray	119	0	119
Total	706	15	691

**Table 2:** Descriptive scope of the four analytical domains.

Domain	Reported Summary	Interpretation Permitted in This Study
ERP	Signed mean voltage in fixed latency windows	Event-locked waveform/window profile
Sustained spectral	Theta/alpha/beta power divided by 1–30 Hz power	ROI- and band-resolved sustained profile
Stage-resolved spectral	Relative power in early/middle/late segment thirds	Within-segment descriptive trajectory
Hilbert band-window	Baseline-relative power change and phase consistency	Exploratory band-window summary

## Data Availability

Data supporting the findings of this study are available from the corresponding author upon reasonable request, subject to ethical and institutional restrictions.

## Supplementary Materials

The following supporting information can be downloaded at: https://www.mdpi.com/article/10.3390/s26185808/s1, Supplementary Methods S1. Exploratory participant-level statistical analysis; Table S1. Analysis-family summary of the omnibus Friedman tests; Table S2. Three lowest uncorrected omnibus p values; none were FDR-significant; Table S3. Accompanying statistical result files and SHA-256 checksums; Table S4. Participant-level source inputs and SHA-256 checksums; Table S5. Complete omnibus Friedman results; Supplementary Data S1. Trial accounting and contributor denominators; Table S6. Event and exclusion accounting; Table S7. Distribution of valid epochs across participant-condition-stage cells; Table S8. Contributor-denominator summary for power and phase consistency; Table S9. Accompanying Supplementary Data S1 files; Figure S1. Condition-by-ROI heatmaps of fixed-band Hilbert baseline-relative power change and phase consistency for theta (4–8 Hz), alpha (8–13 Hz), and beta (13–30 Hz). Values are descriptive means. Phase-consistency cells reflect only one to three available epochs per participant/event cell and are undefined for one-epoch cells; the panels are not full time–frequency decompositions. Absolute phase-consistency magnitude is not evidence of meaningful phase locking, is not quantitatively comparable with conventional many-trial ITC, and was not shown to be stable. For each ROI and band, panels average available participant-stage values across both event stages: power uses 40 values per condition, and phase consistency uses 37 for bedroom-green and 40 for each other condition, from 20 participants. These pooled participant-stage values are not independent participants. Exact denominators are provided in Supplementary Data S1.
