# Supplementary material for: Exploratory EEG Feature Profiles During Viewing of Six Combined Indoor Spatial-Color Conditions
Source: Sensors (Basel). 2026 Sep 14;26(18):5808. doi: 10.3390/s26185808 (PMC13610997; doi:10.3390/s26185808)
Supplement: Supplementary file 1 [file sensors-26-05808-s001.zip › sensors-4474584-supplementary.pdf]

Supplementary Materials

Exploratory EEG Feature Profiles during Viewing of Six Combined Indoor Spatial–Color Conditions

Yingchun Li[1], Jiaxin Zhao[1,2], Le Ren[1], Rui Hong[1], Lingxiao Xi[1], Li Zhang[1] and Xupeng Wang[1,2]

[1] School of Art and Design, Xi'an University of Technology, Xi'an 710054, China

[2] School of Mechanical and Precision Instrument Engineering, Xi'an University of Technology, Xi'an 710048, China

* Correspondence: wangxupeng@xaut.edu.cn (X.W.)

This supplement contains:

- Supplementary Methods S1. Exploratory participant-level statistical analysis
- Table S1. Analysis-family summary of the omnibus Friedman tests.
- Table S2. Three lowest uncorrected omnibus p values; none were FDR-significant.
- Table S3. Accompanying statistical result files and SHA-256 checksums.
- Table S4. Participant-level source inputs and SHA-256 checksums.
- Table S5. Complete omnibus Friedman results.
- Supplementary Data S1. Trial accounting and contributor denominators
- Table S6. Event and exclusion accounting.
- Table S7. Distribution of valid epochs across participant-condition-stage cells.
- Table S8. Contributor-denominator summary for power and phase consistency.
- Table S9. Accompanying Supplementary Data S1 files.
- Figure S1. Condition-by-ROI heatmaps of fixed-band Hilbert baseline-relative power change and phase consistency for theta (4–8 Hz), alpha (8–13 Hz), and beta (13–30 Hz). Values are descriptive means. Phase-consistency cells reflect only one to three available epochs per participant/event cell and are undefined for one-epoch cells; the panels are not full time–frequency decompositions. Absolute phase-consistency magnitude is not evidence of meaningful phase locking, is not quantitatively comparable with conventional many-trial ITC, and was not shown to be stable. For each ROI and band, panels average available participant-stage values across both event stages: power uses 40 values per condition, and phase consistency uses 37 for bedroom-green and 40 for each other condition, from 20 participants. These pooled participant-stage values are not independent participants. Exact denominators are provided in Supplementary Data S1.

Supplementary Methods S1. Exploratory participant-level statistical analysis

Scope notice. This document packages a previously executed exploratory reanalysis. The primary manuscript remains descriptive and does not use these inferential results to support condition-effect claims.

Purpose and analytical boundary

This supplementary analysis was assembled to show what participant-level inferential assessment was performed after reviewer concern about the absence of statistical testing. It is not incorporated into the primary article because the present experimental design, sample, trial structure, and resulting evidence remain insufficient for strong condition-effect claims. The analysis does not isolate color, room function, material, layout, furnishing, or semantic effects because the six stimuli are compound, non-factorial scenes.

Methods

The participant was the inferential unit. The three runs had already been averaged within participant, combined condition, event stage, region of interest, and feature in the authoritative subject-level tables. Each selected endpoint compared the six within-subject conditions with a Friedman test. Kendall's W was used as the omnibus effect-size measure. Benjamini-Hochberg false-discovery-rate correction was applied separately within five analysis families. Pairwise Wilcoxon signed-rank tests were planned only after an FDR-significant omnibus result, followed by within-endpoint BH-FDR correction. Tests were two-sided with alpha = .05.

The five families comprised eight primary ERP endpoints, nine sustained relative-power endpoints, nine selected temporal relative-power endpoints, two Hilbert-power endpoints, and two phase-consistency sensitivity endpoints. Run and presentation sequence were not modeled separately. The phase-consistency analysis excluded one-epoch cells before participant-level aggregation because phase consistency is undefined for a single epoch.

Results

Thirty omnibus Friedman tests were completed. No omnibus result survived within-family BH-FDR correction at $q < .05$. Because no endpoint passed the prespecified omnibus gate, no pairwise Wilcoxon comparison was generated. The two nominal uncorrected p values below .05 must not be interpreted as statistically supported condition effects.

Table S1. Analysis-family summary of the omnibus Friedman tests.

Analysis family	Omnibus tests	FDR-significant
ERP primary	8	0
Sustained relative power	9	0
Temporal selected relative power	9	0
Hilbert power	2	0
Phase consistency sensitivity	2	0

Table S2. Three lowest uncorrected omnibus p values; none were FDR-significant.

Endpoint	n	Friedman chi-square	p	q	Kendall W
Temporal Early Occipital Theta relative power	20	13.486	0.019	0.173	0.135
Temporal Late Parietal Beta relative power	20	11.257	0.047	0.209	0.113
Initial FrontoCentral LPP mean amplitude	20	10.543	0.061	0.490	0.105

Interpretation and limitations

The corrected results do not support post hoc condition rankings, superiority claims, or a claim that any combined condition produced a systematic neural effect. Lack of an FDR-significant result is not evidence of equivalence or absence of an effect. The sample contains 20 young adults; repeated runs do not increase the independent participant sample. The compound stimulus design prevents factor-specific attribution, and participant-level aggregation means that run and sequence effects were not estimated.

All primary ERP, sustained-power, temporal-power, and Hilbert-power endpoints had complete six-condition data for 20

participants. The final phase-consistency sensitivity endpoint had 17 complete participants because P16-P18 had one-epoch Bedroom-Green final-view cells. This endpoint is secondary and does not resolve the broader low-trial stability problem.

Future work should use a larger prospectively planned sample, a factorial stimulus design, more artifact-free trials, prespecified primary endpoints, and a repeated-measures or mixed-effects framework with multiplicity control, effect sizes, uncertainty intervals, and assumption and sensitivity checks.

Accompanying result files

The accompanying CSV files provide all 30 omnibus tests, condition-level descriptive statistics (mean, SD, SE, median, IQR, and t-based 95% confidence intervals), endpoint-level inclusion checks, family summaries, and the header-only post hoc table documenting that no gated pairwise test was run.

Table S3. Accompanying statistical result files and SHA-256 checksums.

File	SHA-256
omnibus_friedman_results.csv	F1778A9B804B4FE51AE9216F146800EB3DB9533F13951600D3BDBCF75F898878
descriptive_statistics.csv	AC72C9963EFA681E94508F2334DECF977725C2AB46C4B333825F6300D9C165D2
analysis_inclusion_qc.csv	254E0D49EC008015D430020E1DC690619CC4FE8394D1ACC3C20190A4802491EF
family_summary.csv	591FA48370DCC1C2D69B249C488E46C3365712A486F3E1FA3A40800A990DFEBB
posthoc_wilcoxon_results.csv	F604C26068082FF643B0A05F6C6E41375AD2E58D6A193C71086532E6AE5C2EF9

Input provenance

The analysis used four existing participant-level feature exports. Their hashes were reverified when this supplement was assembled; the source data themselves are not redistributed in this review package.

Table S4. Participant-level source inputs and SHA-256 checksums.

Source table role	SHA-256
ERP subject-level table	0DEE854463792C35336B690D99A0E26A905E8F260D373DB6570C533FCBB44D8F
Sustained-power subject-level table	B400AF3D073E3BF59E08C9A15071C4005468AC478683F46FB4257D646C3F144D
Temporal-power subject-level table	1AD21C6098680D2F48C10B004F4AD6B1C8F8D2472A0BAA6F7B675D5726EB20ED
Hilbert subject-level table	C24295AF74684D7FB7F3A9C5AECB0CB18CE68CB7734898A1C7EA006A17D73FA5

Table S5. Complete omnibus Friedman results.

Family	Endpoint	n	chi-square	p	q (BH-FDR)	Kendall W
ERP primary	Initial Occipital P1 mean amplitude	20	4.886	0.430	0.874	0.049
ERP primary	Initial Occipital N1 mean amplitude	20	1.714	0.887	0.887	0.017
ERP primary	Initial Parietal P3 mean amplitude	20	3.886	0.566	0.887	0.039
ERP primary	Initial FrontoCentral LPP mean amplitude	20	10.543	0.061	0.490	0.105
ERP primary	Final Occipital P1 mean amplitude	20	4.829	0.437	0.874	0.048
ERP primary	Final Occipital N1 mean amplitude	20	1.971	0.853	0.887	0.020
ERP primary	Final Parietal P3 mean amplitude	20	2.857	0.722	0.887	0.029
ERP primary	Final FrontoCentral LPP mean amplitude	20	5.257	0.385	0.874	0.053
Sustained relative power	Segment Occipital Theta relative power	20	7.829	0.166	0.747	0.078
Sustained relative power	Segment Occipital Alpha relative power	20	4.629	0.463	0.905	0.046
Sustained relative power	Segment Occipital Beta relative power	20	2.029	0.845	0.905	0.020
Sustained relative power	Segment Parietal Theta relative power	20	3.486	0.626	0.905	0.035
Sustained relative power	Segment Parietal Alpha relative power	20	1.629	0.898	0.905	0.016
Sustained relative power	Segment Parietal Beta relative power	20	7.971	0.158	0.747	0.080
Sustained relative power	Segment FrontoCentral Theta relative power	20	1.571	0.905	0.905	0.016
Sustained relative power	Segment FrontoCentral Alpha relative power	20	3.657	0.600	0.905	0.037
Sustained relative power	Segment FrontoCentral Beta relative power	20	2.829	0.726	0.905	0.028
Temporal selected relative power	Temporal Early Occipital Theta relative power	20	13.486	0.019	0.173	0.135
Temporal selected relative power	Temporal Middle Occipital Theta relative power	20	6.714	0.243	0.546	0.067
Temporal selected relative power	Temporal Late Occipital Theta relative power	20	4.086	0.537	0.806	0.041
Temporal selected relative power	Temporal Early Occipital Alpha relative power	20	1.200	0.945	0.945	0.012

Family	Endpoint	n	chi-square	p	q (BH-FDR)	Kendall W
Temporal selected relative power	Temporal Middle Occipital Alpha relative power	20	8.314	0.140	0.419	0.083
Temporal selected relative power	Temporal Late Occipital Alpha relative power	20	4.486	0.482	0.806	0.045
Temporal selected relative power	Temporal Early Parietal Beta relative power	20	1.829	0.872	0.945	0.018
Temporal selected relative power	Temporal Middle Parietal Beta relative power	20	2.971	0.704	0.906	0.030
Temporal selected relative power	Temporal Late Parietal Beta relative power	20	11.257	0.047	0.209	0.113
Hilbert power	Hilbert power Initial global mean dB	20	7.114	0.212	0.425	0.071
Hilbert power	Hilbert power Final global mean dB	20	1.400	0.924	0.924	0.014
Phase consistency sensitivity	Phase consistency Initial global mean	20	2.743	0.740	0.740	0.027
Phase consistency sensitivity	Phase consistency Final global mean	17	4.933	0.424	0.740	0.058

Supplementary Data S1. Trial accounting and contributor denominators

This supplement documents the retained event-locked epochs underlying the manuscript's fixed-band Hilbert power-change and phase-consistency summaries. It tabulates the archived processing records; it does not report a new EEG preprocessing run, new inferential tests, or additional observations.

Participant identifiers P01–P20 are the analysis identifiers. Conditions are B1 bedroom-yellow, B2 bedroom-wood-tone, B3 bedroom-green, L1 living-room-red, L2 living-room-blue, and L3 living-room-gray. Initial and Final denote the two event stages. EventCode F1–F12 represents a position within each participant's assigned sequence, not a fixed condition across participants. The participant-specific mapping is included in S1a and S1e. The same trial count applies to each of the three ROIs and three bands within a participant-condition-stage cell.

Event and exclusion accounting

The 720 planned event positions yielded 719 recorded F-marker occurrences. Removing eight earlier duplicate occurrences, while retaining the last occurrence, left 711 unique markers. Nine planned markers were missing. Five present markers could not form a complete −200-to-800-ms epoch at the recording boundary. Of the 706 epochable events, 15 were rejected by the executed automated criterion: absolute voltage exceeding 150 µV at any sample in any retained EEG channel within the complete epoch. This left 691 clean epochs. Retention is 691/706 = 97.9%; the denominator is epochable events, not planned positions. No manual epoch rejection was performed in this processing branch.

S1e distinguishes missing markers, boundary-incomplete epochs, amplitude rejection, and retained epochs. EarlierDuplicatesRemoved is recorded separately and is not an additional planned event. TotalEpochs means epochable events, and TotalEpochs = CleanEpochs + RejectedEpochs. For each S1a cell, PlannedRuns = MissingMarkers + BoundaryIncomplete + Epochable, and Epochable = RejectedEpochs + ValidEpochs.

Table S6. Event and exclusion accounting.

Accounting item	Count or value
Planned event positions	720
Recorded F-marker occurrences	719
Earlier duplicate occurrences removed	8
Unique markers after duplicate resolution	711
Missing planned markers	9
Boundary-incomplete present markers	5
Epochable events	706
Amplitude-rejected epochs	15
Clean epochs	691
Retention among epochable events	97.9% (691/706)
Manual epoch rejection	0

Of 240 participant-condition-stage cells, 214 had three valid epochs, 23 had two, and three had one. The three one-epoch cells were P16, P17, and P18 in bedroom-green Final (F12). Their phase-consistency values were undefined and omitted, not replaced by zero. Power change remained defined for these cells. Stage-specific phase means therefore have 17 contributors for bedroom-green Final and 20 for every other condition-stage combination; power means have 20 throughout. These exclusions do not remove the participants from other conditions, event stages, or feature domains.

Table S7. Distribution of valid epochs across participant-condition-stage cells.

Valid epochs per cell	Number of cells	Share of 240 cells
3	214	89.2%
2	23	9.6%
1	3	1.3%

Valid epochs per cell	Number of cells	Share of 240 cells
Total	240	100.0%

Executed aggregation rule

For each participant and mapped condition/event stage, channels were averaged within each ROI. The retained epochs from contributing runs were collected as separate epoch columns; continuous recordings were not concatenated in time. A fourth-order Butterworth band-pass filter was applied forward and backward separately to each epoch, followed by its Hilbert analytic signal. At each time sample, phase consistency was the magnitude of the mean unit phasor across epochs. This magnitude was then averaged over the band-specific window: theta 4–8 Hz at 200–600 ms, alpha 8–13 Hz at 200–800 ms, and beta 13–30 Hz at 300–800 ms. The measure was undefined when fewer than two epochs contributed. It was not computed by averaging single-run ITC values.

Instantaneous power was expressed in dB relative to each epoch's −200-to-0-ms baseline and then averaged over the band-specific window and retained epochs. Stage-specific group summaries are unweighted means of available participant-level values, omitting undefined phase values. S1c reports the resulting denominators separately for power and phase consistency.

The existing Figure S1 pools available participant-stage feature values across Initial and Final, matching the archived figure-generation procedure. Each ROI/band/condition power panel mean uses 40 participant-stage values. Phase panel means use 37 for bedroom-green and 40 for each other condition, all drawn from 20 participants. These 37 or 40 pooled values are not independent participants. S1d reports both the denominators and the unchanged means underlying this aggregation.

Table S8. Contributor-denominator summary for power and phase consistency.

Summary	Denominator	Participant base
Stage-specific power, all condition-stage cells	20 participant values	20
Stage-specific phase, bedroom-green Final	17 participant values	17
Stage-specific phase, all other condition-stage cells	20 participant values	20
Figure S1 power, each condition pooled across stages	40 participant-stage values	20
Figure S1 phase, bedroom-green pooled across stages	37 participant-stage values	20
Figure S1 phase, each other condition pooled across stages	40 participant-stage values	20

Provenance and limitations

The ledger reconciles the archived event-cleanup records, per-run epoch-rejection summaries, participant-specific event mapping, selected-run index, and featureonly_ersp_itc_subject_avg_mapped.csv from the final raw-rerun processing branch. The algorithm was checked against run_feature_only_raw_rerun.m; Figure S1 aggregation was checked against make_feature_only_nature_figures.py. Recomputed descriptive means and contributor counts agree with descriptive_ersp_itc_features.csv. The author's source records and existing figure pixels were not modified.

Exact accounting improves transparency but cannot establish reliability from one to three epochs. Absolute phase-consistency magnitude is not evidence of meaningful neural phase locking, is not quantitatively comparable with conventional many-trial ITC, and does not justify condition ranking or a mechanistic interpretation. This supplement remains descriptive technical documentation.

Table S9. Accompanying Supplementary Data S1 files.

File	Rows	Unit	SHA-256
S1a_trial_counts_by_participant_condition_stage.csv	240	Participant × combined condition × event stage	333228CE312BF0A35D1385D26A640335BD59A9095 C59B8C77DA2745F023C110A
S1b_trial_count_distribution.csv	12	Combined condition × event stage	57A1062B7843FA0E87C8E594EE954FA61006C50A4D 35516FCE155521128FBEFB
S1c_phase_power_contributor_denominators.csv	108	Combined condition × event stage × ROI × band	063E7ABF05F6968DF85E993F6951E1A3EE2AA1E50 794FD8A796561F473F6079B
S1d_figure_S1_collapsed_denominators.csv	54	Combined condition × ROI × band, pooled across event stages	C5EBB2B7138A7CF778081D2DD8F2C257104D7D9A 86CFEC85815EC7E10B02B3F6
S1e_event_disposition_by_run.csv	720	Planned participant × run × event position	4FBB09B6419D3FFBAF30F0EE1F0B35C7FD9EA8D4 9DE1A19FC8FFFAC9867CE5BB

Supplementary Figure

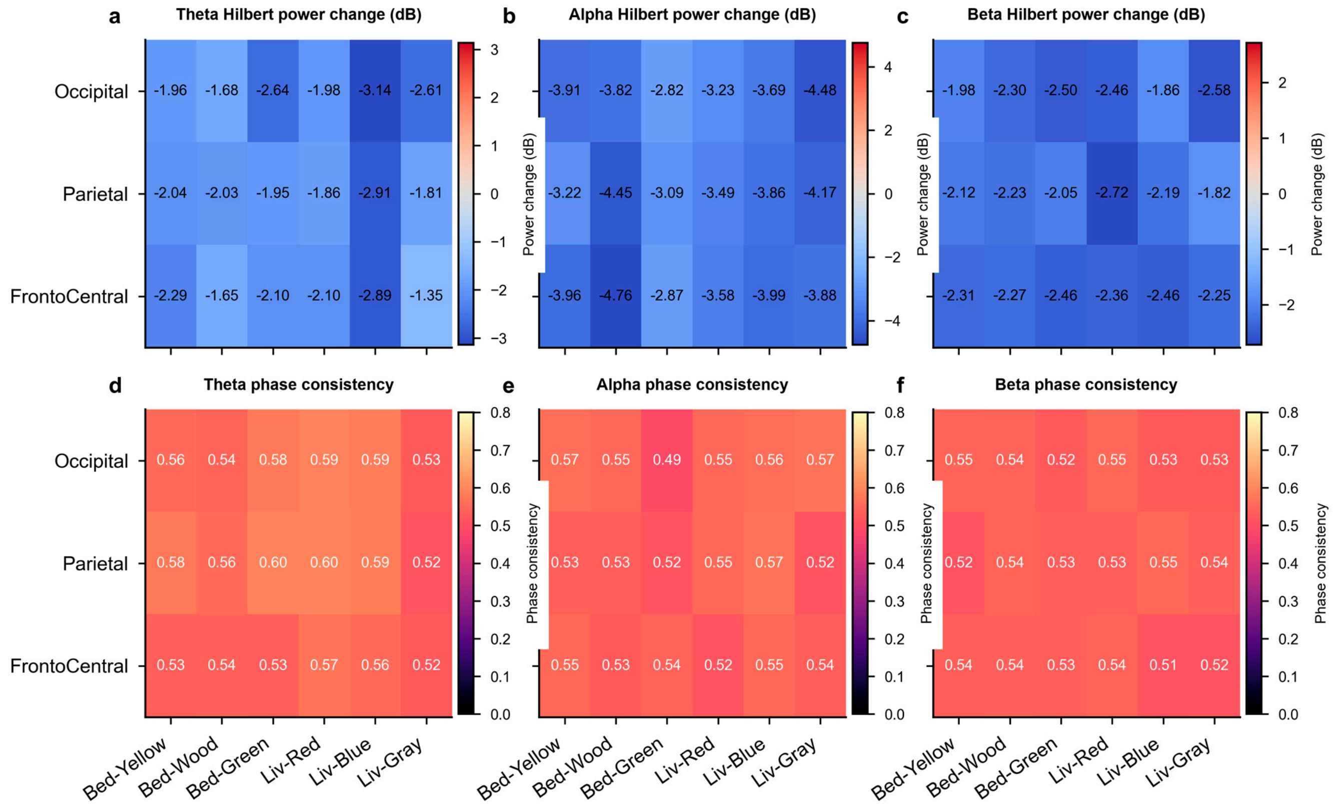

Figure S1. Condition-by-ROI heatmaps of fixed-band Hilbert baseline-relative power change and phase consistency for theta (4–8 Hz), alpha (8–13 Hz), and beta (13–30 Hz). Values are descriptive means. Phase-consistency cells reflect only one to three available epochs per participant/event cell and are undefined for one-epoch cells; the panels are not full time–frequency decompositions. Absolute phase-consistency magnitude is not evidence of meaningful phase locking, is not quantitatively comparable with conventional many-trial ITC, and was not shown to be stable. For each ROI and band, panels average available participant-stage values across both event stages: power uses 40 values per condition, and phase consistency uses 37 for bedroom-green and 40 for each other condition, from 20 participants. These pooled participant-stage values are not independent participants. Exact denominators are provided in Supplementary Data S1.
